# Transcriptional Changes in the Developing Rice Seeds Under Salt Stress Suggest Targets for Manipulating Seed Quality

**DOI:** 10.3389/fpls.2021.748273

**Published:** 2021-11-08

**Authors:** Choonseok Lee, Chong-Tae Chung, Woo-Jong Hong, Yang-Seok Lee, Jong-Hee Lee, Hee-Jong Koh, Ki-Hong Jung

**Affiliations:** ^1^Graduate School of Biotechnology and Crop Biotech Institute, Kyung Hee University, Yongin, South Korea; ^2^Crop Research Division, Chungcheongnam-do Agricultural Research and Extension Services, Yesan, South Korea; ^3^School of Life Sciences, University of Warwick, Coventry, United Kingdom; ^4^Department of Southern Area Crop Science, National Institute of Crop Science, Miryang, South Korea; ^5^Department of Agriculture, Forestry and Bioresources, Research Institute for Agriculture and Life Sciences, and Plant Genomics and Breeding Institute, Seoul National University, Seoul, South Korea

**Keywords:** climate change, *Oryza sativa*, reclaimed land, salt stress, seed quality, sea-level rise, transcriptional changes in developing seeds

## Abstract

Global sea-level rise, the effect of climate change, poses a serious threat to rice production owing to saltwater intrusion and the accompanying increase in salt concentration. The reclaimed lands, comprising 22.1% of rice production in Korea, now face the crisis of global sea-level rise and a continuous increase in salt concentration. Here, we investigated the relationship between the decrease in seed quality and the transcriptional changes that occur in the developing rice seeds under salt stress. Compared to cultivation on normal land, the japonica rice cultivar, Samgwang, grown on reclaimed land showed a greatly increased accumulation of minerals, including sodium, magnesium, potassium, and sulfur, in seeds and a reduced yield, delayed heading, decreased thousand grain weight, and decreased palatability and amylose content. Samgwang showed phenotypical sensitivity to salt stress in the developing seeds. Using RNA-seq technology, we therefore carried out a comparative transcriptome analysis of the developing seeds grown on reclaimed and normal lands. In the biological process category, gene ontology enrichment analysis revealed that the upregulated genes were closely associated with the metabolism of biomolecules, including amino acids, carboxylic acid, lignin, trehalose, polysaccharide, and chitin, and to stress responses. MapMan analysis revealed the involvement of upregulated genes in the biosynthetic pathways of abscisic acid and melatonin and the relationship of trehalose, raffinose, and maltose with osmotic stress. Interestingly, many seed storage protein genes encoding glutelins and prolamins were upregulated in the developing seeds under salt stress, indicating the negative effect of the increase of storage proteins on palatability. Transcription factors upregulated in the developing seeds under salt stress included, in particular, bHLH, MYB, zinc finger, and heat shock factor, which could act as potential targets for the manipulation of seed quality under salt stress. Our study aims to develop a useful reference for elucidating the relationship between seed response mechanisms and decreased seed quality under salt stress, providing potential strategies for the improvement of seed quality under salt stress.

## Introduction

During the 20th century, global sea-levels rose by approximately 12–22cm. Satellite images reveal that the sea-levels are currently rising by approximately 3.4mm per year (Walthall et al., 2012). Sea-level rise is affected mainly by climate change (Mimura, 2013), and the global average surface temperature has increased by approximately 0.74°C (0.56°C–0.92°C; Walthall et al., 2012). Between 1955 and 2010, the world ocean heat content also continuously increased (Mimura, 2013), and ocean temperatures as well as sea-levels are predicted to continue to rise indefinitely (Walthall et al., 2012). Sea-level rise directly affects the quality of water available on lands and in rivers near the coast, and the World Bank has predicted a reduction in rice production in Thailand and Vietnam, both rice exporters, due to an increase in the salinification of key river deltas caused by sea-level rise (Walthall et al., 2012). Around 80,000 hectares on the Gulf Coast in the United States are utilized for rice production; however, because of long-term subsidence, saltwater has intruded into rice production areas (Walthall et al., 2012). In the Republic of Korea (Korea), 81 reclaimed lands (RLs) were prepared mostly in southern and western coastal areas, comprising a total area of 186,639 hectares, corresponding to 11.7% of the total arable land and 22.1% of the land used for rice production (Lim et al., 2020). RLs in Korea, although critically important for rice production, are extremely vulnerable to sea-level rise and its accompanying increase in salt concentration because compared to normal land (NL), they already contain a significantly higher salt concentration and are located near the sea (Walthall et al., 2012; Lim et al., 2020). Accordingly, RLs are highly effective test sites for determining the effect of sea-level rise on rice cultivation and seed harvest.

Several studies of rice cultivation and production have reported that salt stress causes abnormal agronomic traits, resulting in a decrease in yield (Cheong et al., 1995; Choi et al., 2003; Baxter et al., 2011; Thitisaksakul et al., 2015), thousand grain weight (Peiris et al., 1988; Choi et al., 2003; Thitisaksakul et al., 2015), and a delay of heading (Cheong et al., 1995; Choi et al., 2003). Under salt stress, particularly, sodium (Na; Peiris et al., 1988; Cheong et al., 1995) and protein (Peiris et al., 1988; Choi et al., 2003; Thitisaksakul et al., 2015) contents were increased compared with those under normal conditions without salt stress. Moreover, Choi et al. (2003) reported that salt stress decreased the palatability of rice seeds. In rice seeds, the increase in protein content resulted in a decrease in eating quality (Juliano et al., 1965). Furthermore, the seed quality of rice is among the most important agronomic traits in rice breeding programs (Lau et al., 2015; Kobayashi et al., 2018).

Therefore, we focused on the abnormalities observed in the agronomic trait of seed quality under salt stress. To understand the decrease in seed quality of rice under salt stress during its entire life cycle, we investigated how transcriptional changes occur in the developing rice seeds under salt stress through RNA-sequencing. To date, investigations on the global transcriptome changes occurring in the developing rice seeds under salt stress are limited. Therefore, we aimed to provide a useful reference for exploring molecular mechanisms to improve or maintain the quality of rice seeds under salt stress.

## Materials and Methods

### Growth of Rice Plants and Investigation of Agronomic Traits

In 2018, we transplanted Samgwang seedlings (*Oryza sativa* subsp. *japonica*) and cultivated them on NL, the experimental paddy field of the Chungcheongnam-do Agricultural Research and Extension Service (CARES, latitude: 36.74°N, longitude: 126.82°E), and RL, the Seosan reclaimed land Section B (latitude: 36.66°N, longitude: 126.34°E). We cultivated three replicates by a completely randomized design using the standard rice cultivation method of CARES (Supplementary Figure 1). We further investigated agronomic traits, including days until heading, culm length, panicle length, spikelet numbers per panicle, thousand grain weight, and yield. Mature seeds were harvested at 146days after transplanting from 100 plants per replicate.

### Analysis of the Chemical Properties of Topsoil Samples Collected From NL and RL

We collected topsoil samples from NL and RL in three replicates and sent them to the National Instrumentation Center for Environmental Management (NICEM), Seoul, Republic of Korea, for chemical analysis. The NICEM, using its in-house methods, performed the following chemical analyses: pH; electrical conductivity (EC); and total nitrogen (N), phosphorus (P), sodium (Na), potassium (K), magnesium (Mg), calcium (Ca), and sulfur (S) contents. Based on the summary of the analysis methods performed in the NICEM, we analyzed pH and EC using a pH meter (HM-30R, DKK-TOA, Japan) and an EC meter (CM-25R, DKK-TOA, Japan), respectively. In addition, we analyzed the total nitrogen content using a Kjeltec 2400/8400 Kjeldahl Analyzer (FOSS Tecator AB, Sweden) and quantified P, Na, K, Mg, Ca, and S using inductively coupled plasma atomic emission spectroscopy (ICP-1000IV, Shimadzu, Japan; An et al., 2018; Lim and Yi, 2019).

### Investigation of Palatability and Amylose, Total Nitrogen, P, Na, K, Mg, Ca, S, and Trehalose Contents in the Rice Seeds of Samgwang Grown on NL and RL

We analyzed the palatability and amylose, N, P, Mg, K, Ca, and S contents of the brown or milled rice of Samgwang, a japonica rice cultivar, at the Department of Southern Area Crop Science, National Institute of Crop Science. We examined the palatability of the Samgwang seeds grown and harvested on NL and RL as per the method described by Lee et al. (2020a) using a TOYO mido meter (MA-90B, TOYO, Japan). All analyses were performed for two of the three replicates owing to limited research funds. We measured amylose content in milled rice using Juliano’s method (Juliano, 1971) and quantified total nitrogen (N) in brown rice using the Macro Determinator (CN928, LECO Corp., U.S.A.) using Lee’s methods (Lee et al., 2020a). We analyzed the P, K, Mg, Ca, and S contents of brown rice through an energy dispersive X-ray fluorescence spectrometer (NEX CG, Applied Rigaku Technologies, Inc., U.S.A.) using the methods published in the journal, Plant Breeding and Biotechnology (Lee et al., 2020b).

We quantified sodium (Na) content in brown rice using inductively coupled plasma atomic emission spectroscopy (ICP-1000IV, Shimadzu, Japan) according to the methods presented by Lim and Yi (2019).

We quantified trehalose content in brown rice using the methods developed in the Center for University-Wide Research Facilities at the Jeonbuk National University, which employs liquid chromatography/mass spectrometry–mass spectrometry (LC/MS-MS; Kim et al., 2021). We purchased standard trehalose from Sigma-Aldrich (Saint Louis, MO, United States).

### RNA-Sequencing of the Developing Seeds of Samgwang Grown on NL and RL Through Illumina Sequencing and Further Analyses of RNA-Sequencing Data

We collected the developing seeds of Samgwang grown on NL and RL in two replicates. The developing seeds were sampled from one panicle per plant and six panicles per replicate at 8 and 15days after heading (DAH), respectively. Next, we extracted total RNA from frozen and ground samples of the developing seeds using the RNeasy Plant Mini Kit (QIAGEN, Hilden, Germany) according to the manufacturer’s instructions. We sent the total RNA samples to Macrogen, Inc (Seoul, Republic of Korea) for RNA-seq using the Illumina technology (the RNA-seq raw data obtained by paired-end type sequencing with 101-bp read length are available at: https://www.ebi.ac.uk/arrayexpress/experiments/E-MTAB-10774).

We carried out the cleaning procedure for raw sequence reads by removing low-quality nuculeotides (Phred score<20) and short sequence reads (read length<20nt). Next, we mapped the processed reads on the Nipponbare reference genome sequence obtained from the Rice Genome Annotation Project Database^1^ (version 7.0) using the HISAT2 software with default parameters (Kim et al., 2019a). Furthermore, we quantified the raw read counts in the BAM file using featureCounts software (Liao et al., 2013). We also conducted an analysis of differentially expressed genes (DEGs) using the DESeq2 package (Love et al., 2014) and selected genes with *p*<0.05 and |Log_2_(fold change)| of >1 (i.e., |fold change|>2).

Gene ontology (GO) enrichment was performed according to the method described by Hong et al. (2021). We retrieved GO terms for the biological function of DEGs from the Rice Oligo Array Database.^2^ The enrichment value was calculated by dividing the query total with the query expected value, and we chose GO terms having a Hyper value of *p*<0.05 and an enrichment value of >2 (Hong et al., 2021). For the functional classification of DEGs, we used MapMan software to group genes into distinct functional categories and visualize the data through various overview diagrams. Finally, we uploaded the RGAP Locus IDs to MapMan to obtain their functional classifications (Usadel et al., 2009).

### Gene Expression Analysis by Quantitative Real Time-Polymerase Chain Reaction

We synthesized cDNA from 1μg of total RNA using the iScript^™^ cDNA Synthesis Kit (Bio-Rad, Hercules, CA, United States). The primer sets used for qRT-PCR are presented in Supplementary Table 1. We used the rice actin gene (*OsACT*; *LOC_Os11g06390*) as a reference gene (Supplementary Table 1). We performed qRT-PCR in the Rotor-Gene Q system (QIAGEN, Hilden, Germany) using iQ SYBR Green Supermix (Bio-Rad, Hercules, CA, United States). In addition, we determined the relative expression of genes presented in Supplementary Table 1 using the Pfaffl method (Pfaffl, 2004; Kim et al., 2019b; Moon et al., 2019).

### Generation of Heatmap

We generated the heatmap data to graphically express DEGs detected in developing rice seeds under salt stress using Mev software^3^ (Moon et al., 2020).

### Statistical Analysis

We performed the *t*-test and correlation analysis using SAS 9.4 TS Level 1M5 (Ver.1.0.19041; SAS Institute Inc., Cary, NC, United States).

## Results

### Topsoils Collected From NL and RL Showed Different Chemical Properties

Both NL (the experimental paddy field of CARES) and RL (section B of the Seosan reclaimed land) used for field experiments were located in Chungcheongnam-do, Republic of Korea, and share very similar latitudes (Supplementary Figure 1). We compared the chemical properties of the topsoil samples collected from NL and RL (Table 1). Of the chemical properties analyzed, we found no significant differences in terms of pH and P content between the two locations. N and Ca contents in RL were significantly lower than those in NL; however, other minerals, such as Na, K, Mg, and S, and EC in RL were significantly higher than those in NL. The topsoil of RL exhibited significantly high amount of Na, while that of NL exhibited scant amounts of Na (Table 1).

**Table 1:** The chemical properties of topsoil for NL and RL, respectively, in 2018.

Location	pH	EC (ds/m)	N (%)	P (mg/kg)	Na (mg/kg)	Mg (mg/kg)	K (mg/kg)	S (mg/kg)	Ca (mg/kg)
NL	5.2 ± 0.36	0.35 ± 0.038	0.142 ± 0.0067	15.85 ± 3.62	46.03 ± 9.71	97.22 ± 21.78	108.98 ± 17.21	267.61 ± 2.67	856.96 ± 149.83
RL	5.2 ± 0.20	4.17 ± 0.787	0.127 ± 0.0038	13.62 ± 0.31	1344.45 ± 198.65	330.52 ± 35.71	286.17 ± 34.41	1460.23 ± 137.56	302.74 ± 54.18
Values of *p*^#^	1.0000	0.0011	0.0246	0.3473	0.0003	0.0006	0.0013	0.0001	0.0038

*Data represent mean±standard deviation (SD)*.

# *The value of p for each chemical property was determined by analysis of t-test between NL and RL*.

### Rice Grains Developing Under Salt Stress Exhibited Reduced Seed Quality and Enhanced Na^+^ Content

**Figure 1:**
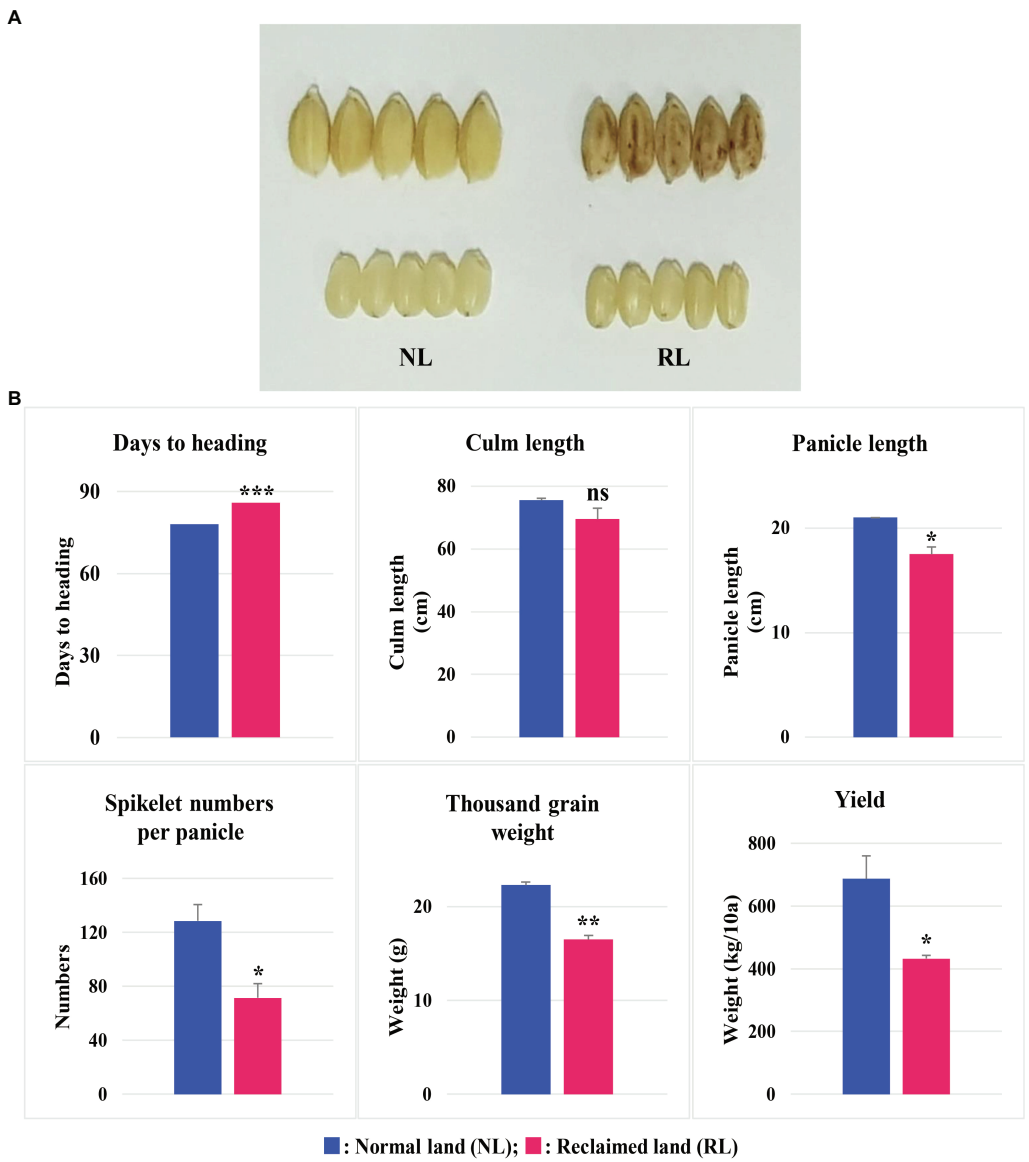
Agronomic traits of Samgwang grown on normal land (NL) and reclaimed land (RL) in 2018. **(A)** Rice seeds harvested on NL and RL. **(B)** Agronomic traits, including days until heading, culm length, panicle length, spikelet numbers per panicle, thousand grain weight, and yield. Data represent mean±SD. ns: not significant; ^*^0.01<*p*<0.05; ^**^0.001<*p*<0.01; ^***^p<0.001. Significant difference for each dataset was determined by *t*-test.

We selected Samgwang, a japonica rice cultivar, with high palatability for this study, because Samgwang is one of the 18 premium rice cultivars registered in Korea and has the largest cultivation area in Chungcheongnam-do (Cho et al., 2020). We then examined the agronomic traits of Samgwang, grown on NL and RL (Figure 1) and confirmed the apparent damage caused by salt stress in unhulled and brown rice of Samgwang grown on RL, but not in those on NL. The heading date of Samgwang was significantly delayed on RL compared to that on NL, and the thousand grain weight and yield were decreased more significantly on RL than that on NL. However, no significant difference in culm length between NL and RL was observed in Samgwang. In addition, Samgwang exhibited a significant difference in terms of panicle length and spikelet numbers per panicle on RL, compared to those on NL (Figure 1).

We further analyzed the quality of seeds of Samgwang harvested on both NL and RL by using palatability and amylose, N, and mineral contents, including P, Na, K, Mg, and S contents (Table 2). Of these minerals, seeds of Samgwang grown on NL contained only a small amount of Na, but those grown on RL accumulated huge amounts of Na. For seeds of Samgwang grown on RL compared to those grown on NL, amylose content and palatability were significantly decreased, and N contents was significantly higher in Samgwang seeds grown on RL, but no significant difference between NL and RL was observed in P contents on Samgwang seeds. However, the seeds of Samgwang grown on RL exhibited a significant increase in the accumulation of minerals, including Na, K, Mg, and S compared to those grown on NL (Table 2).

**Table 2:** Palatability, amylose, total nitrogen and minerals, including P, Na, Mg, K and S, analyzed from mature seeds of Samgwang harvested in NL and RL in 2018.

Location	Palatability	Amylose (%)	N (%)	P(mg/kg)	Na (mg/kg)	Mg (mg/kg)	K (mg/kg)	S (mg/kg)
NL	76.9 ± 0.6	18.01 ± 0.06	1.16 ± 0.05	2830.0 ± 438.4	17.73 ± 1.07	2380.0 ± 70.7	3085.0 ± 106.1	901.0 ± 1.4
RL	67.8 ± 1.1	14.75 ± 0.22	1.81 ± 0.01	3737.5 ± 208.6	729.28 ± 77.93	3227.5 ± 194.5	3,525 ± 77.8	1390.0 ± 42.4
Value of *p*^#^	0.0095	0.0025	0.0031	0.1183	0.0059	0.0285	0.0419	0.0037

*Data represent mean±SD*.

# *The value of p for each chemical property was determined by analysis of t-test between NL and RL*.

### Transcriptional Changes in the Rice Developing Seeds Under Salt Stress

**Figure 2:**
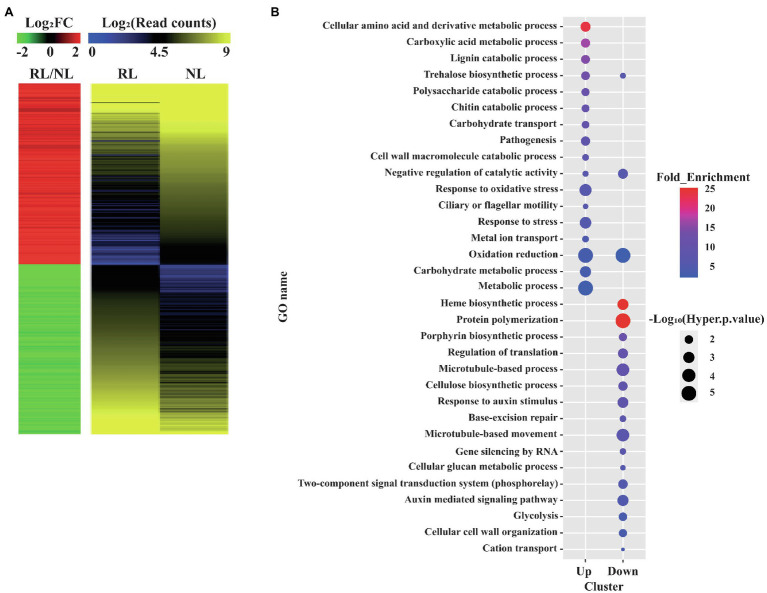
Gene ontology (GO) enrichment analysis of differentially expressed genes (DEGs) detected in the developing seeds of Samgwang grown on reclaimed land (RL). **(A)** Heatmap analysis of the DEGs. A total of 491 up or downregulated DEGs between normal land (NL) and RL were visualized. Both heatmaps were generated by using average values of four replicates in the transcriptome analysis. The red and green colors in the left panel indicate up and downregulated DEGs, respectively. The Log_2_FC (FC, fold change) and Log_2_(Read counts) indicate the Log_2_ transformed value of normalized read counts and fold change (RL/NL), respectively, which was generated by RNA-seq. **(B)** GO enrichment analysis of the 491 DEGs. GO terms based on the biological process were visualized as the dot plot. Dot color and size represent fold enrichment value (query number/query expected value in selected GO term) and−Log_10_ (Hyper value of *p*). The redder and larger dots indicate statistically significant enriched GO terms.

We performed RNA-seq of Samgwang using the total RNA of developing seeds at 8 and 15 DAH by Illumina sequencing for the investigation of the transcriptional changes occurring in the developing seeds under salt stress using RNA-seq analysis. After the raw data of RNA-seq for the developing seeds of Samgwang were obtained, we combined the raw data obtained for the developing seeds grown on NL and RL at 8 and 15 DAH. The objective was to obtain universal DEGs throughout seed development with four biological replicates. We obtained DEGs from further analysis of raw data (see “Materials and methods”; Figure 2A; Supplementary Table 2). We selected DEGs under both two-fold change and adjusted value of *p* <0.05, thereby arriving at the selection of 253 upregulated and 238 downregulated DEGs (Figure 2A; Supplementary Table 2). To confirm the reliability of DEGs generated from the combined raw data, correlation analysis was then performed for the expression values of DEGs obtained at 8 and 15 DAH seeds grown on NL and RL (Supplementary Table 3). For seeds grown on NL and RL, the expression values of DEGs at 8 DAH highly correlated with those at 15 DAH (i.e., 0.91802, *p*<0.0001 for seeds grown on NL and 0.94980, *p*<0.0001 for seeds grown on RL), indicating that reproducibility between biological replicates obtained at 8 and 15 DAH for each treatment is very high (Supplementary Table 3).

### GO Enrichment Analysis Suggests a Close Association Between Salt Stress During the Seed Ripening Stage and Metabolism and Stress Responses

We performed GO enrichment analysis to classify DEGs into GO terms corresponding to the biological process (Figure 2B). We found upregulated DEGs under salt stress to be closely associated with metabolisms of several biomolecule-related GO terms, including the cellular amino acid and derivative metabolic process (GO:0006519), the carboxylic acid metabolic process (GO:0019752), the lignin catabolic process (GO:0046274), the trehalose biosynthetic process (GO:0005992), the polysaccharide catabolic process (GO:0000272), and the chitin catabolic process (GO:0006032) as well as stress-related GO terms, including response to oxidative stress (GO:0006979) and response to stress (GO:0006950). DEGs downregulated under salt stress were closely associated with GO terms different from those of DEGs upregulated under salt stress, and included protein polymerization (GO:0051258), the cellulose biosynthetic process (GO:0030244), the regulation of translation (GO:0006417), base-excision repair (GO:0006284), the microtubule-based process (GO:0007017), and the negative regulation of catalytic activity (GO:0043086; Figure 2B).

### Significance of Abscisic Acid, Melatonin, and Carbohydrate Biosynthesis Pathways in Seed Development Under Salt Stress Was Emphasized Through MapMan Analysis and Verified by qRT-PCR, Supporting the Reliability of RNA-Seq Data

**Figure 3:**
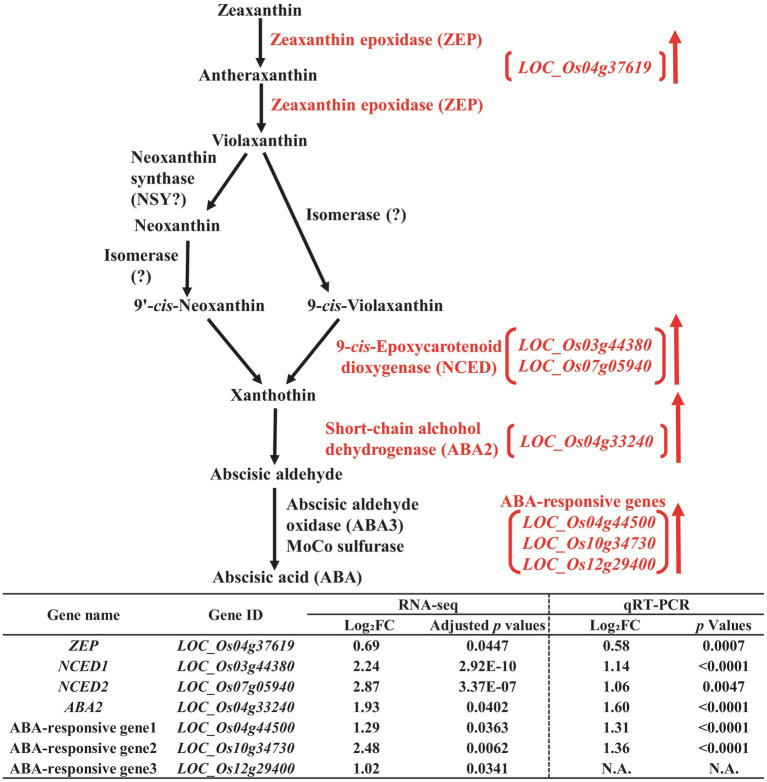
Biosynthetic pathway of abscisic acid (ABA) in the developing seeds of Samgwang grown on reclaimed land (RL) and verification of upregulated genes through qRT-PCR. ↑ indicates upregulation. The Log_2_FC (FC, fold change) expresses the Log_2_ value of a ratio of expression value of RL to that of normal land (NL; RL/NL), which was generated by RNA-seq and qRT-PCR, respectively, from the developing seeds of Samgwang. The significant difference for each dataset was determined by *t*-test.

**Figure 4:**
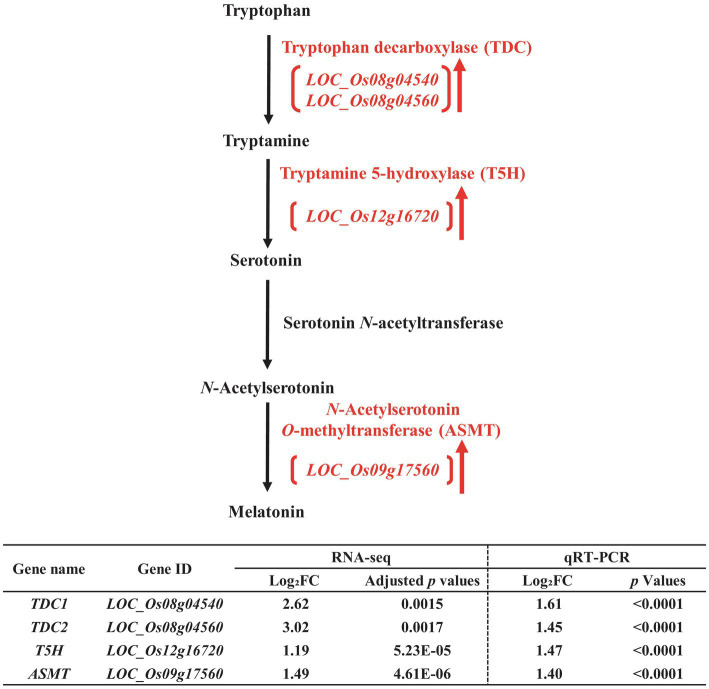
Biosynthetic pathway of melatonin in the developing seeds of Samgwang grown on reclaimed land (RL) and verification of upregulated genes through qRT-PCR. **↑** indicates upregulation. The Log_2_FC (FC, fold change) expresses the Log_2_ value of a ratio of expression value of RL to that of normal land (NL; RL/NL), which was generated by RNA-seq and qRT-PCR, respectively, from the developing seeds of Samgwang. The significant difference for each dataset was determined by *t*-test.

**Figure 5:**
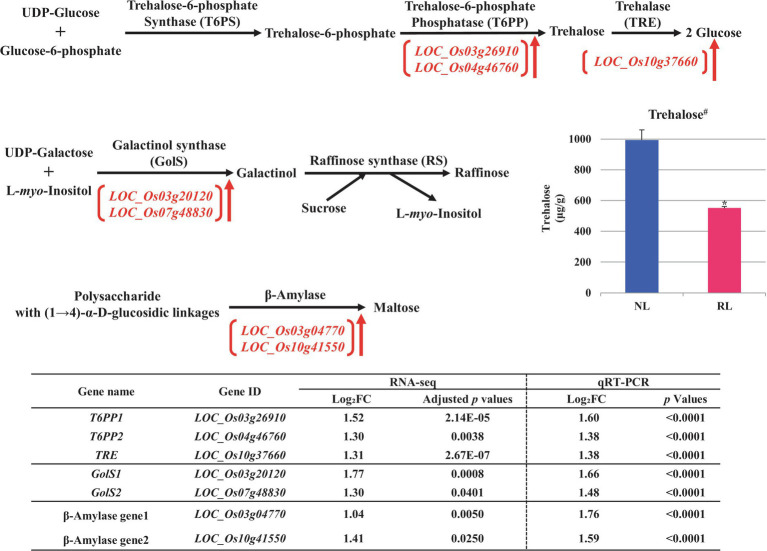
Metabolic pathways of carbohydrates putatively related to salt stress in the developing seeds of Samgwang grown on reclaimed land (RL) and verification of upregulated genes through qRT-PCR. **(A)** Trehalose; **(B)** Raffinose; **(C)** Maltose. ↑ indicates upregulation. ^#^indicates that trehalose was quantified in the brown rice of Samgwang grown on normal land (NL) and RL by LC/MS–MS. The Log_2_FC (FC, fold change) expresses the Log_2_ value of a ratio of expression value of RL to that of NL (RL/NL), which was generated by RNA-seq and qRT-PCR, respectively, from the developing seeds of Samgwang. The significant difference for each dataset was determined by *t*-test. ^*^0.01<*p*<0.05.

To perform additional functional classification of the selected DEGs found in developing rice seeds in response to salt stress, we performed MapMan analysis (Supplementary Figure 1). Our data from MapMan analysis confirmed the putative relationship to drought and/or salt stress of DEGs involved in the biosynthetic pathways of biomolecules—including ABA (Mizrahi et al., 1971; Most, 1971; Arad et al., 1973), melatonin (Mukherjee et al., 2014), trehalose (Garcia et al., 1997; Vasquez-Robinet et al., 2008), raffinose (Pattanagul and Madore, 1999; Bellaloui et al., 2013), and maltose (Fougère et al., 1991; Rizhsky et al., 2004), and we therefore chose these biomolecules for further analysis (Figures 3–5).

We detected 9-cis-Epoxycarotenoid dioxygenase (*NCED*, *LOC_Os03g44380* and *LOC_Os07g05940*; Qin and Zeevaart, 1999) and short-chain alcohol dehydrogenase (*ABA2*, *LOC_Os04g33240*; Schwartz et al., 1997), involved in the biosynthetic pathway of ABA, as DEGS upregulated by salt stress (Figure 3) in addition to ABA-responsive genes, including *LOC_Os04g44500*, *LOC_Os10g34730*, and *LOC_Os12g29400*. Additionally, zeaxanthin epoxidase (*ZEP*, *LOC_Os04g37619*), although not included in DEGs with 2-fold change, did however exhibit an approximately 1.6-fold change with adjusted value of *p* <0.05, indicating its trend toward a high salt stress response. Next, we performed qRT-PCR to verify RNA-seq data for the aforementioned genes through relative expression data from qRT-PCR for all genes except for *LOC_Os12g29400*, one of the ABA-responsive genes, due to difficulties in the design of the proper primer set for PCR reaction (Figure 3).

**Figure 6:**
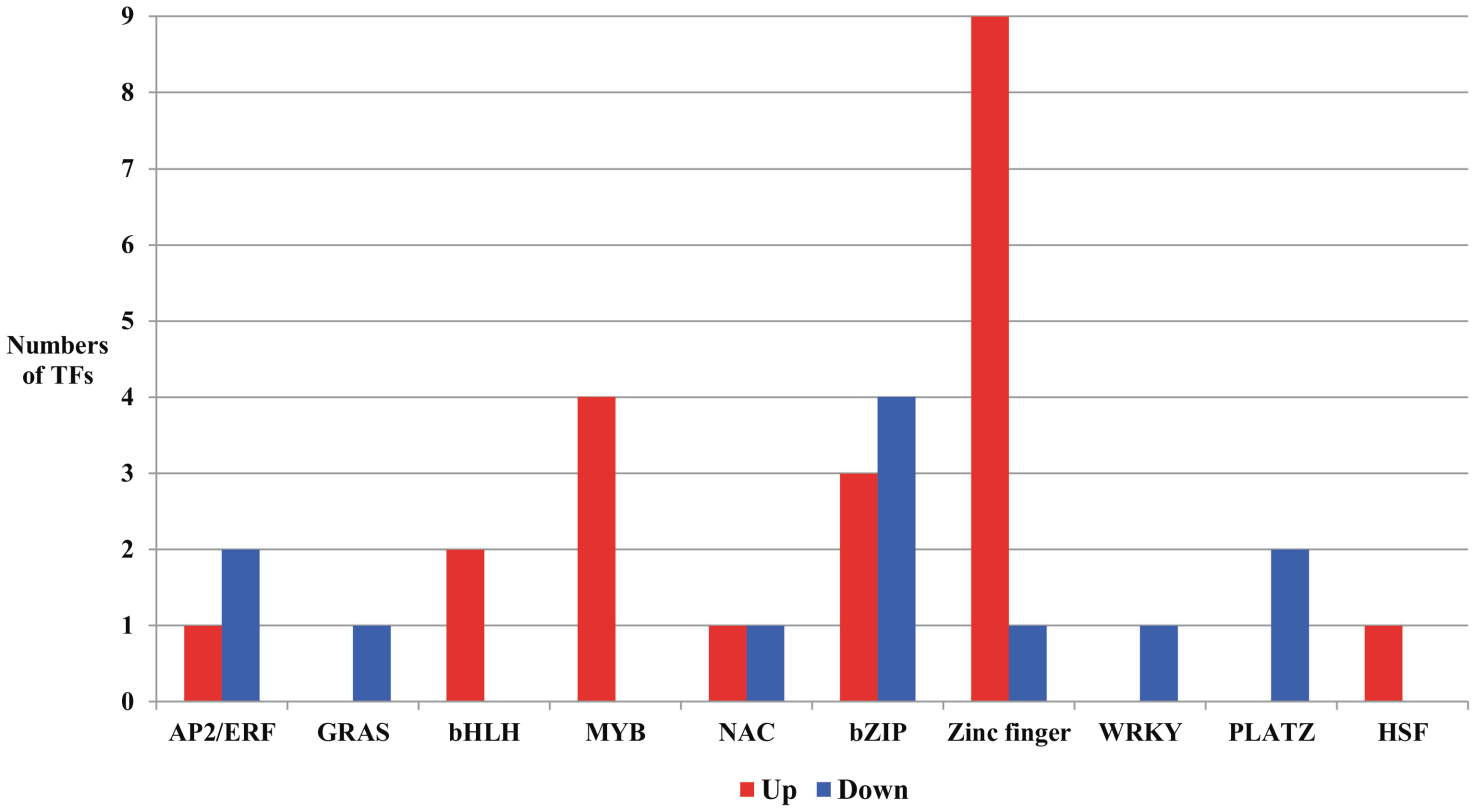
Transcription factors (TFs) up or downregulated due to salt stress in the developing seeds of Samgwang. AP2/ERF: APETALA2/ethylene responsive factor; GRAS: GAI (Gibberellin-Acid-Insensitive), RGA (Repressor of GA1), SCL (SCARECROW); bHLH, basic helix-loop-helix; NAC: NAM, ATAF, and CUC; bZIP, basic region/leucine zipper; PLATZ, plant AT-rich sequence and zinc binding; HSF, heat shock factor.

Similar to the biosynthetic pathway of ABA, the biosynthetic pathway of melatonin was also upregulated under salt stress (Figure 4). In Figure 6 we present upregulated DEGs, including tryptophan decarboxylase (*TDC*, *LOC_Os08g04540* and *LOC_Os08g04560*; Noé et al., 1984; De Luca et al., 1989; Songstad et al., 1990), tryptamine 5-hydroxylase (*T5H*, *LOC_Os12g16720*; Kang et al., 2007; Fujiwara et al., 2010), and N-acetylserotonin O-methyltransferase (*ASMT*, *LOC_Os09g17560*; Kang et al., 2011). We also verified these DEGs through qRT-PCR, thereby confirming the corresponding RNA-seq data (Figure 4).

In terms of the metabolic pathways of several carbohydrates, including trehalose, raffinose, and maltose, related to drought and/or salt stress, we confirmed the salt stress-induced upregulation of DEGs including trehalose-6-phosphate phosphatase (*T6PP*, *LOC_Os26910* and *LOC_Os04g46760*), trehalase (*TRE*, *LOC_Os10g37660*; Müller et al., 2001), galactinol synthase (*GolS*, *LOC_Os03g20120* and *LOC_Os07g48830*; Taji et al., 2002), and β-amylase (*LOC_Os03g04770* and *LOC_Os10g41550*; Figure 5). Furthermore, we performed the verification of these selected DEGs through qRT-PCR, to confirm their correspondence with RNA-seq data.

### LC/MS–MS Analysis Reveals the Reduction of Trehalose in Seeds Grown on RL

Interestingly, of the above carbohydrates, *T6PP* (*LOC_Os26910* and *LOC_Os04g46760*) and *TRE* (*LOC_Os10g37660*), both involved in the metabolic pathway of trehalose, catalyze trehalose 6-phosphate into trehalose and convert trehalose into glucose, respectively (Figure 5A). We analyzed the trehalose content of the brown rice of the japonica rice cultivar, Samgwang, grown on NL and RL using LC/MS–MS to investigate whether the quantity of trehalose corresponded to the expression of *T6PP* (*LOC_Os26910* and *LOC_Os04g46760*) and *TRE* (*LOC_Os10g37660*). Our results indicated that the quantity of trehalose was significantly decreased in the brown rice of the cultivars grown on RL compared to that of those grown on NL, explaining seed damage caused by salt stress in RL (Figures 1, 5).

### Transcriptional Regulation and the Response of Storage Proteins in Developing Seeds Under Salt Stress Are Important

From DEGs in developing seeds of Samgwang both up- and downregulated under salt stress, we searched transcription factors (TFs) to unearth more transcriptional regulation for responses of developing rice seeds by salt stress (Figure 6; Supplementary Table 4). We categorized 33 TFs detected in DEGs data as AP2/ERF (APETALA2/Ethylene Responsive Factor; Licausi et al., 2013), GRAS [GAI(Gibberellin-Acid-Insensitive), RGA(Repressor of GA1), SCL(SCARECROW); Pysh et al., 1999], bHLH (basic helix-loop-helix; Hu et al., 1996; Kiribuchi et al., 2004, 2005), MYB (Dai et al., 2007), NAC (NAM, ATAF, and CUC; Ohnishi et al., 2005; Hu et al., 2006), basic region/leucine zipper (bZIP; Kusano et al., 1995; Gupta et al., 1998), zinc finger (von Arnim and Deng, 1993; Lippuner et al., 1996; Song et al., 1998), WRKY (Zhang et al., 2004; Wei et al., 2008), plant AT-rich sequence and zinc binding (PLATZ; Wang et al., 2018, 2019), and heat shock factor (HSF; Hübel et al., 1995; Lee et al., 1995; Xiang et al., 2013). In developing rice seeds under salt stress, DEGs for GRAS, WRKY, and PLATZ were downregulated while DEGs for bHLH, MYB, and HSF were upregulated. Furthermore, in AP2/ERF, NAC, bZIP, and zinc finger, we identified genes showing both up- and down-regulation. Specifically, for zinc finger, we detected a total of ten DEGs in developing rice seeds under salt stress, nine of which were upregulated. Interestingly, in GO analysis data (biological process) of upregulated DEGs for TFs, two bZIPs (*LOC_Os02g43330* and *LOC_Os09g21180*) and two zinc fingers (*LOC_Os02g52210* and *LOC_Os09g26780*) were grouped into “response to stress,” and two bHLHs (*LOC_Os02g45170* and *LOC_Os09g28210*) and one NAC (LOC_Os05g34310) were classified as “anatomical morphogenesis” and “multicellular organismal development,” respectively. All DEGs for MYB, including *LOC_Os01g09640*, *LOC_Os01g64360*, *LOC_Os01g74410*, and *LOC_Os04g42950*, were categorized by the program as “biosynthetic process” (Figure 6; Supplementary Table 4).

In addition, we found, unsurprisingly, that genes for seed storage proteins, including glutelin and prolamin, were upregulated by salt stress (Supplementary Table 5). DEGs for seed storage proteins comprise 10 glutelin genes and 17 prolamin genes, indicating that seed storage proteins might be affected by osmotic stress mediated by salinity.

We further generated DEGs dataset for the developing seeds of Samgwang at 8 and 15 DAH, respectively, to exhibit the information of DEGs according to the seed developmental stages (Supplementary Table 6). Under salt stress, DEGs from 8 DAH seeds of Samgwang contained 460 upregulated and 335 downregulated genes, while, in 15 DAH seeds of Samgwang, 243 upregulated and 98 downregulated genes were detected (Supplementary Table 6). Several transcription factors, including NAC (*LOC_Os05g34310*), HSF (*LOC_Os08g43334*), bZIP (*LOC_02g43330*, *LOC_Os07g08420* and *LOC_Os09g21180*) and zinc finger (*LOC_Os03g49730* and *LOC_Os09g26780*), were selected in Figure 6; Supplementary Table 4 to verify newly generated DEGs data of the developing seeds of Samgwang at 8 and 15 DAH, respectively, using qRT-PCR (Supplementary Table 7). The expression patterns of tested transcription factor genes detected at 8 and/or 15 DAH seeds through RNA-seq are well matched with qRT-PCR data, but some genes showed significant expression at 8 or 15 DAH in the qRT-PCR data in spite of no expression at a specific developmental stage in the RNA-seq data (Supplementary Table 7). For further confirmation, 5 heat shock protein genes detected in DEGs data of the developing seeds of Samgwang at 8 and 15 DAH, respectively, were selected (Supplementary Tables 6, 8). As expected, the RNA-seq data correspond to the data of qRT-PCR, with the exception for some genes at a specific developmental stage (Supplementary Table 8). These results indicate that DEGs data from each developmental stage of Samgwang seeds might be useful for further studies.

## Discussion

Global sea-level rise, which is an effect of climate change, has continued to occur throughout the entire 20th century, and is currently ongoing, resulting in saltwater intrusion (Walthall et al., 2012; Mimura, 2013). The RLs, corresponding to 11.7% of the total arable land and 22.1% of that used for rice production in Korea face a crisis of sea-level rise, which directly causes an increase in salt concentrations (Mimura, 2013; Lim et al., 2020). As reported by Cheong et al. (1995), topsoil samples of some selected RLs in the western coastal areas of Korea contain significantly higher levels of minerals, including Na, K, and Mg, than those of NLs, apart from the Ca levels, which are higher in topsoils of NL, as shown in Table 1. Calcium deficiency is known to occur in alkaline soils. However, little is known about Ca deficiency in the topsoils of RL (Bower and Turk, 1946). In plants, Ca functions as a structural component that stabilizes the cell wall, a secondary messenger of physiological, developmental, and stress-related processes, and also as an element in plant immune signaling (Thor, 2019). Regarding the growth and development of plants, the relatively different Ca content between NL and RL is a promising topic for further research.

The growth of rice plants of Samgwang, the japonica rice cultivars, was negatively affected by a high salt condition, i.e., among the agronomic traits examined in this study, salt stress significantly decreased yield and thousand grain weight and significantly delayed heading date (Figure 1B); this was consistent with the results of previous studies (Peiris et al., 1988; Cheong et al., 1995; Choi et al., 2003; Baxter et al., 2011; Thitisaksakul et al., 2015). Similar to the research data reported by Peiris et al. (1988) and Cheong et al. (1995), we confirmed a significant accumulation of minerals such as Na, K, Mg, and, S in the seeds of Samgwang grown on RL (Table 2), which was caused by saline soil containing considerably higher levels of several minerals than normal soil (Table 1). Under salinity, however, palatability and amylose content were significantly decreased in the seeds of Samgwang (Table 2), indicating a decrease in seed quality as shown by Choi et al. (2003). Moreover, it is still largely unknown how the mineral content of soil is related to the accumulation of minerals in seeds and various agronomic traits. Therefore, further investigation is necessary to determine the mechanism underlying abnormal agronomic traits related to the transcription of genes in developing rice seeds and provide a better understanding of the effect of salinity on rice seeds, facilitating a more successful rice breeding program to overcome the decrease in seed quality under salt stress. This is of critical importance because, in rice breeding programs, after yield, seed quality is considered to be one of the most important agronomic traits (Lau et al., 2015; Kobayashi et al., 2018).

In plants, the salt overly sensitive (SOS) pathway has been known as the system for the removal of Na^+^ through Ca^2+^ signaling because salt stress causes osmotic stress and the accompanying accumulation of ions, resulting in a decrease in plant growth; however, as we show in Table 2, in RL, minerals—including Na, K, Mg, and S—accumulated significantly in the mature seeds of Samgwang compared to those accumulating in seeds grown on NL (Lamers et al., 2020). The GO enrichment analysis of DEGs, selected from RNA-seq data of the rice developing seeds under salt stress, demonstrated the close association between some upregulated DEGs and stress-related GO terms, including response to oxidative stress (GO:0006979) and response to stress (GO:0006950; Figure 2B). Furthermore, data from our MapMan analysis confirmed that the biosynthetic pathway of ABA was upregulated in the rice developing seeds under salt stress (Figure 3; Supplementary Figure 2; Mizrahi et al., 1971; Most, 1971; Arad et al., 1973). It has been established that ABA regulates osmosis, ions, and reactive oxygen species under salt stress (Lamers et al., 2020). Moreover, salt stress is related to the biosynthesis of melatonin (Zhan et al., 2019; Ahn et al., 2021), which is upregulated by it (Figure 4). As we show in Figure 2B, we identified GO terms related to the metabolic pathways of several biomolecules, and further MapMan analysis exhibited that the biosynthetic pathways of trehalose (Garcia et al., 1997; Vasquez-Robinet et al., 2008), raffinose (Pattanagul and Madore, 1999; Bellaloui et al., 2013), and maltose (Fougère et al., 1991; Rizhsky et al., 2004), all related to drought and/or salt stress, were also upregulated by it (Figure 5; Supplementary Figure 2). The upregulated biosynthesis of these metabolites, related to abiotic stress, could be closely connected to a decrease in both thousand grain weight and amylose content in the seeds of Samgwang (Figure 1; Table 2), resulting in an excessive use of energy to protect their own seeds from osmotic and ionic stress. In addition, the expression of seed storage protein genes, including glutelin and prolamin, showed an increase under salinity (Supplementary Table 5) and, as outlined by Baxter et al. (2011), corresponded to an increase in the glutelin content of rice seeds; however, prolamin content under salinity varies according to each rice cultivar. Interestingly, elevated ozone concentration caused an increase in protein content (Frei et al., 2012; Zhou et al., 2015) and a decrease in yield and starch concentration in rice (Frei et al., 2012), thereby corresponding to our data presented in Figure 1; Table 2; and Supplementary Table 5. This indicates that rice plants might optimize their metabolism in seeds to survive abiotic stress.

We detected TFs involved in transcriptional regulation of responses in developing rice seeds to salt stress, and categorized them as AP2/ERF (Licausi et al., 2013), GRAS (Pysh et al., 1999), bHLH (Hu et al., 1996; Kiribuchi et al., 2004, 2005), MYB (Dai et al., 2007), NAC (Ohnishi et al., 2005; Hu et al., 2006), bZIP (Kusano et al., 1995; Gupta et al., 1998), zinc finger (von Arnim and Deng, 1993; Lippuner et al., 1996; Song et al., 1998), WRKY (Zhang et al., 2004; Wei et al., 2008), PLATZ (Wang et al., 2018, 2019), and HSF (Hübel et al., 1995; Lee et al., 1995; Xiang et al., 2013; Figure 6; Supplementary Table 4). Of these TFs, all bHLHs, MYBs, zinc finger, and HSF genes (apart from one zinc finger gene) were upregulated in developing seeds under salt stress (Figure 6; Supplementary Table 4). Under salt stress, previous studies reported the upregulation of bHLH (*LOC_Os09g28210*; Li et al., 2015), MYB (*LOC_Os01g64360*; Zhou et al., 2016), and zinc finger (*LOC_Os03g60560*; Zhou et al., 2016), but the exact biological functions for these genes are not known (Li et al., 2015; Zhou et al., 2016). Furthermore, the overexpression of *LOC_Os09g26780*—a Zinc finger protein gene—enhanced salt-tolerance in rice (Peethambaran et al., 2018). Drought induced another MYB, *LOC_Os04g42950* (Yu et al., 2020). Salt stress induced bZIP (*LOC_Os02g43330*), an upregulated DEG (Zhou et al., 2016). Interestingly, the overexpression of AP2/ERF (*LOC_Os05g49700*) resulted in a reduction of rice plant height (Ma et al., 2020). The focus of these previous studies was the TFs of shoot and/or root and the whole plant body, but not developing seeds (Li et al., 2015; Zhou et al., 2016; Peethambaran et al., 2018; Ma et al., 2020). This indicates the likelihood that unlike the genes of other organs, such as shoot and root, seed-specific genes are expressed in developing rice seeds.

As noted above, we investigated Samgwang, a premium rice cultivar registered in Korea, with high palatability for a case study of transcriptional changes occurring in developing seeds under salt stress. However, to obtain more robust data concerning the response to high salinity, more transcriptome analysis might be required to examine other rice cultivars with different genetic background, resulting in more reliable scientific data on the response of seeds developing under salt stress.

## Conclusion

Overall, our study represents a case study on the responses of rice plants, focused on seeds, to salt stress. Particularly, we discussed phenotypic and transcriptional changes related to seed quality here. Many remaining questions should be addressed through further studies on the responses of developing rice seeds to salt stress. The maintenance of a sustainable balance between survival under salinity and the improvement of seed quality remains to be addressed through manipulation and a combination of DEGs in rice breeding programs.

## Data Availability Statement

The datasets presented in this study can be found in online repositories. The names of the repository/repositories and accession number(s) can be found in the article/Supplementary Material.

## Author Contributions

CL, H-JK, and K-HJ conceived and designed the experiments. CL analyzed the data. CL, C-TC, W-JH, Y-SL, and J-HL conducted the experiments. CL wrote the manuscript. CL, W-JH, Y-SL, H-JK, and K-HJ contributed to the manuscript revision. All authors contributed to the article and approved the submitted version.

## Funding

This work was supported by grants from the Next-Generation BioGreen 21 Program funded by the Rural Development Administration (no. PJ013165 to H-JK), the Collaborative Genome Program of the Korea Institute of Marine Science and Technology Promotion, funded by the Ministry of Oceans and Fisheries (no.20180430 to K-HJ), and the National Research Foundation, Ministry of Education, Science and Technology (2021R1A2C2010448 to K-HJ).

## Conflict of Interest

The authors declare that the research was conducted in the absence of any commercial or financial relationships that could be construed as a potential conflict of interest.

## Publisher’s Note

All claims expressed in this article are solely those of the authors and do not necessarily represent those of their affiliated organizations, or those of the publisher, the editors and the reviewers. Any product that may be evaluated in this article, or claim that may be made by its manufacturer, is not guaranteed or endorsed by the publisher.

## Supplementary Material

The Supplementary Material for this article can be found online at: https://www.frontiersin.org/articles/10.3389/fpls.2021.748273/full#supplementary-material

## Footnotes

^1^http://rice.plantbiology.msu.edu

^2^http://ricephylogenomics-khu.org/road/go_analysis.php

^3^https://mev.tm4.org

## Abbreviations

ABA: abscisic acid
ABA2: ABA deficient2, short-chain alcohol dehydrogenase
ASMT: N-acetylserotonin O-methyltransferase
GolS: galactinol synthase
NCED: 9-cis-Epoxycarotenoid dioxygenase
TDC: tryptophan decarboxylase
T5H: tryptamine 5-hydroxylase
T6PP: trehalose-6-phosphate phosphatase
TRE: trehalase
ZEP: zeaxanthin epoxidase
AP2/ERF: APETALA2/Ethylene Responsive Factor
bHLH: basic helix–loop–helix
GRAS: GAI (Gibberellin-Acid-Insensitive), RGA (Repressor of GA1), SCL (SCARECROW)
HSF: heat shock factor
NAC: NAM, ATAF, and CUC
PLATZ: plant AT-rich sequence and zinc binding
SOS: salt overly sensitive
