# Supplementary material for: Transcriptional Changes in the Developing Rice Seeds Under Salt Stress Suggest Targets for Manipulating Seed Quality
Source: Front Plant Sci. 2021 Nov 8;12:748273. doi: 10.3389/fpls.2021.748273 (PMC8606889; doi:10.3389/fpls.2021.748273)
Supplement: Supplementary file 1 [file Data_Sheet_1.zip › Data Sheet 1.DOCX]

Supplementary Material


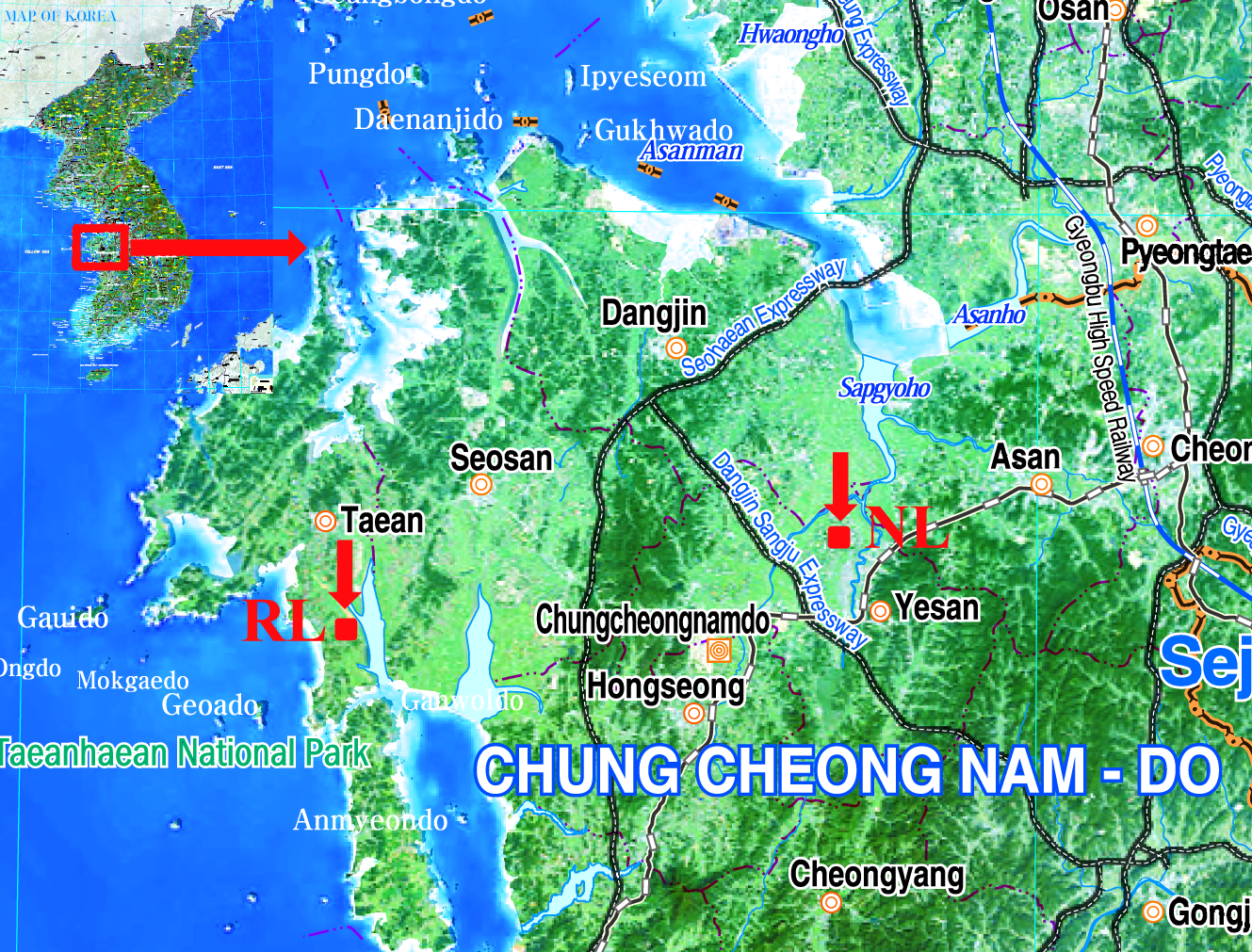


**Supplementary Figure 1.** Locations of field experiments. Normal land (NL), experimental paddy field of CARES (latitude: 36.74°N, longitude: 126.82°E); Reclaimed land (RL), Seosan reclaimed land Section B (latitude: 36.66°N, longitude: 126.34°E). The map of the Republic of Korea was obtained from http://map.ngii.go.kr/mi/emapMain/emapIntro01.do and adapted for the study.


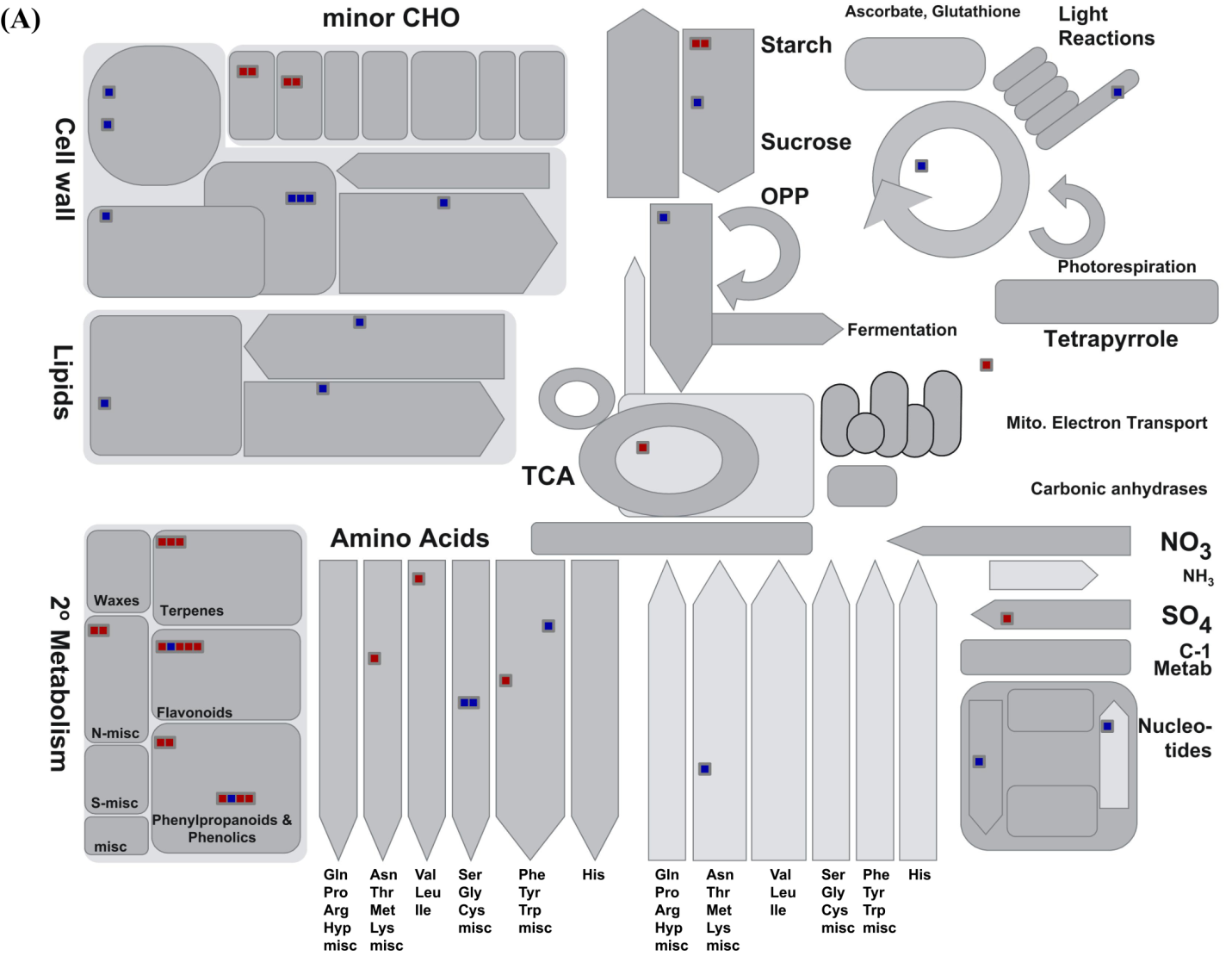


**Up;**

**Down**

**Supplementary Figure 2.** MapMan analysis using differentially expressed genes detected in developing seeds of Samgwang, a japonica rice cultivar, grown in reclaimed land. (A) Metabolism overview; (B) Regulation overview; (C) Cell function overview; (D) Transport overview.


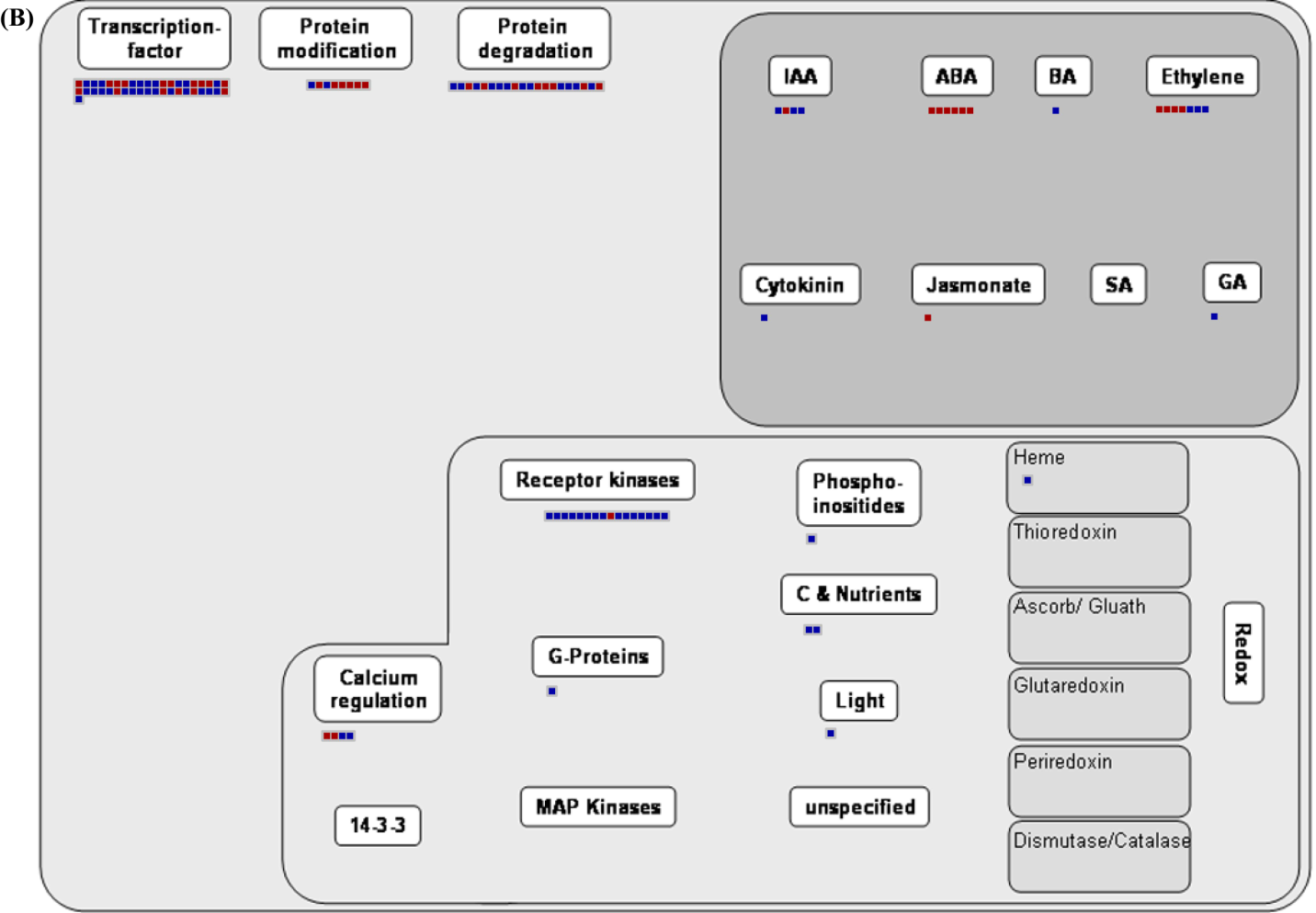


**Up;**

**Down**

**Supplementary Figure 2.** Continued.


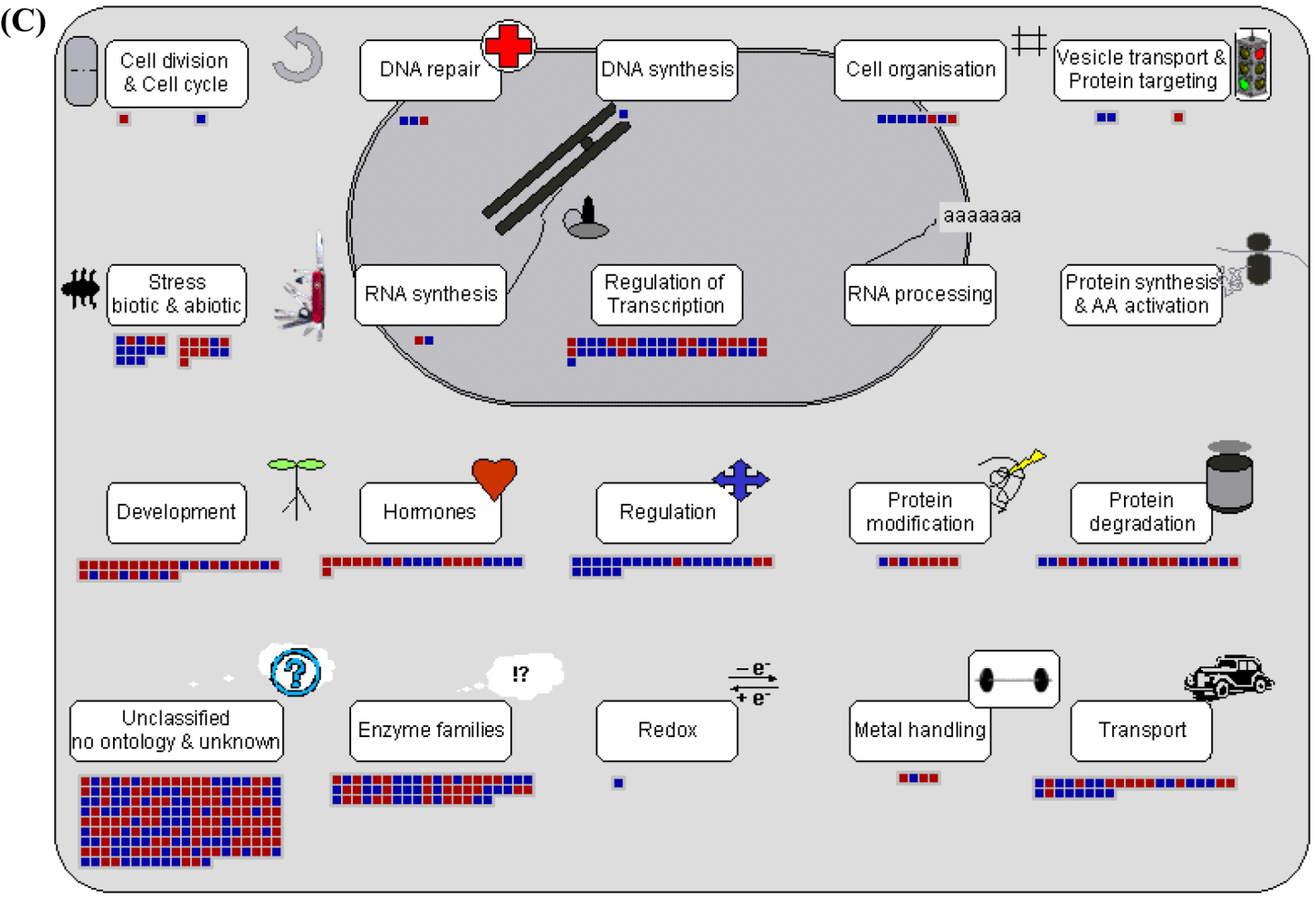


**Up;**

**Down**

**Supplementary Figure 2.** Continued.


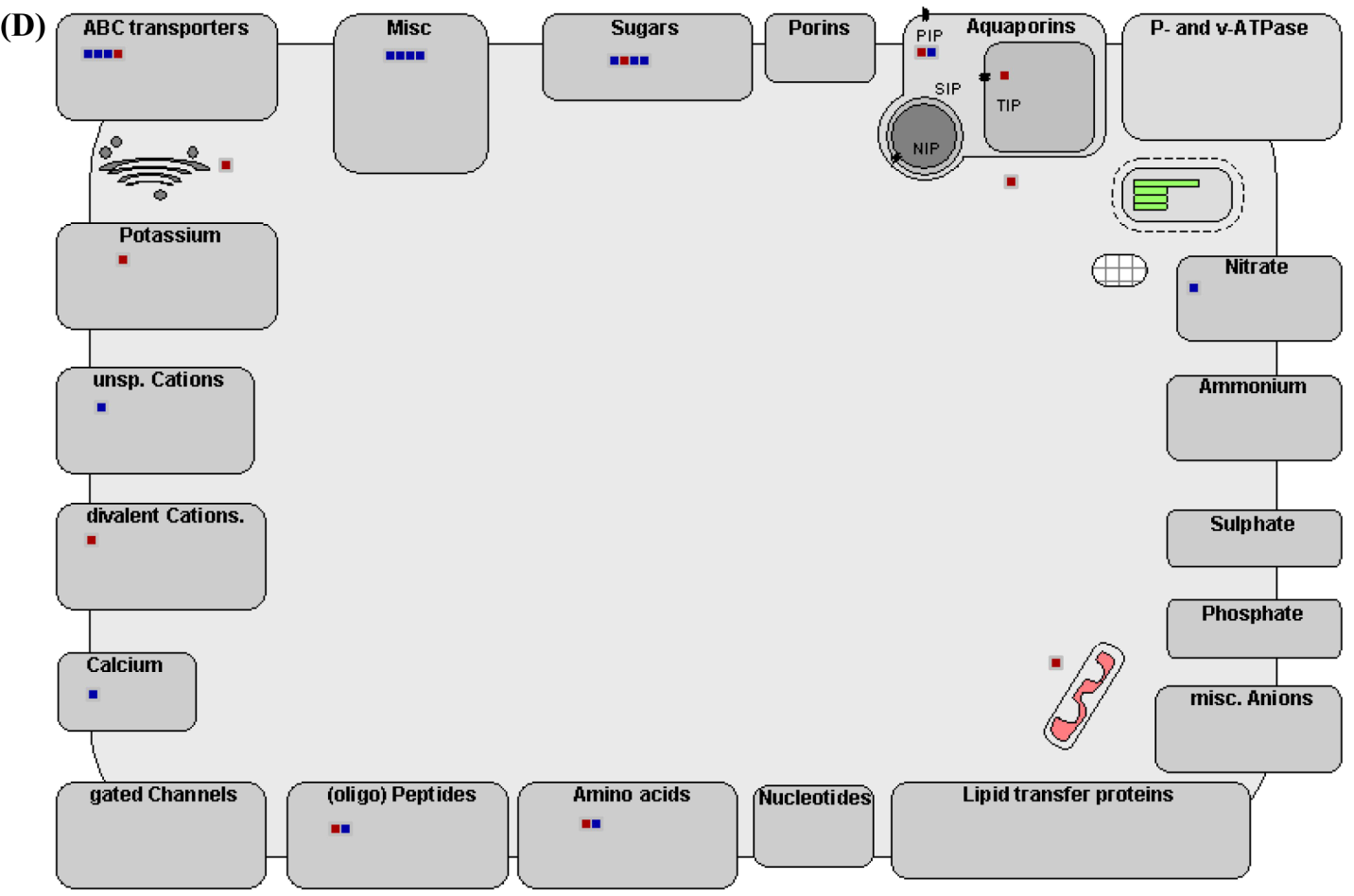


**Up;**

**Down**

**Supplementary Figure 2.** Continued.

**Supplementary Table 1.** Primer sets for quantitative real time-PCR (qRT-PCR).

| Gene name | Gene ID | Product size  (bp) | Forward (5' → 3') | Reverse (5' → 3') | Annealing temperature  (°C) |
| --- | --- | --- | --- | --- | --- |
| *ACT* | *LOC_Os11g06390* | 160 | GGTCCTCTTCCAGCCCTCTT | ACGGTCAGCAATACCAGGGA | 58 |
| *ZEP* | *LOC_Os04g37619* | 250 | GCCACAGATATCAGAAAACCATGC | GATTTGCGGCCCTCCTCTTG | 58 |
| *NCED1* | *LOC_Os03g44380* | 160 | CACCAAGTTCGAGTACGGCG | GGCATTGACGACGAGGAGC | 58 |
| *NCED2* | *LOC_Os07g05940* | 160 | GAGGAGGAGAGCTTCCGCAG | AGGCGTACCGCGTCTTCC | 58 |
| *ABA2* | *LOC_Os04g33240* | 250 | GATCCGCGTCAACTGCGTGT | GAAGATGCCAAAGGACGGGTTG | 58 |
| ABA-responsive gene1 | *LOC_Os04g44500* | 160 | CCCTACAAGGTGGTGGTCCC | GCATGTACTTGCACGACCTCTG | 58 |
| ABA-responsive gene2 | *LOC_Os10g34730* | 160 | AAGGAGGCATAACAAGGGTGCC | GTGTGGCTGATGAACCCCATAA | 58 |
| *TDC1* | *LOC_Os08g04540* | 160 | GGGGCTCAAGCTCTGGATGG | GAAGCAGACGAGAGCGAAGT | 58.5 |
| *TDC2* | *LOC_Os08g04560* | 160 | GGGCTCAAGCTCTGGATGGT | TGAAGCAGACGAGCGCGAAG | 58.5 |
| *T5H* | *LOC_Os12g16720* | 160 | CTCGGCGAGCTCCACTACAT | CCATGGCGAACGTGTTGATGA | 58.5 |
| *ASMT* | *LOC_Os09g17560* | 250 | CGTATTCCCGACGCCATCCA | CAAACACGCGCACCATTTGC | 58 |
| *T6PP1* | *LOC_Os03g26910* | 160 | AACAAGTTCTGCCTCTCCGTCC | CCTTGTCCCACTTGATGGCG | 58.5 |
| *T6PP2* | *LOC_Os04g46760* | 160 | GGGACAAGGGAAAAGCCGTC | ACCTGCGAAACAAGAATCCCAC | 58 |
| *TRE* | *LOC_Os10g37660* | 160 | TATCTCACAGAACCGCGCCA | AGTTGCGATTCCTGCAGGTTG | 58.5 |
| *GolS1* | *LOC_Os03g20120* | 160 | CGGCAAGATGACCGCCAAG | GAGGACGGCGACCACCAG | 58 |
| *GolS2* | *LOC_Os07g48830* | 250 | CCGCAGTACGACATCGGCTA | GAACGAGGTTGTAGACGTTGGG | 58 |
| *BAM1* | *LOC_Os03g04770* | 160 | GGTGCTCAACTTCACGTGCG | CGCCGTCTCGTCGTACCTC | 58 |
| *BAM2* | *LOC_Os10g41550* | 160 | GTACGACTGGGAGGGCTACGG | CGGGTTCGACTTCATCTCCTCCA | 60 |
| *NAC* | *LOC_Os05g34310* | 160 | ACCGTCTCACAAACACGGGA | CGTGAGACGACGTTTGATGGTG | 59 |
| *HSF* | *LOC_Os08g43334* | 160 | TGCAACGGATACGAGTACGACC | ATTCTTGCACAGCCCCCACA | 58 |
| *bZIP1* | *LOC_Os02g43330* | 160 | AAGCGCGCCCGGTGG | CTTGCCGCTCGTTCAGCTTC | 58 |
| *bZIP2* | *LOC_Os07g08420* | 160 | ATGCTCATGCCAAGCAAGCTAC | CGTCTAGCTGACTCCCGGTT | 58 |
| *bZIP3* | *LOC_Os09g21180* | 160 | TCGCCGCCGACAACGAG | CGACCTTCTTCGAGCTGAAACGAC | 59 |
| *Zinc finger1* | *LOC_Os03g49730* | 160 | GGTACTGTGGGAGTGCGTGT | GCCTTGTCATCCTGCAGTTTTCT | 59 |
| *Zinc finger2* | *LOC_Os09g26780* | 160 | CGATCTGCCGATTGCAAGGA | TGTCATCTCTTGGCCAAACCCA | 59 |
| Heat shock protein genes | *LOC_Os01g04380* | 160 | CATCGACTGGAAGGAGACGCC | GCGGTGCCACTTGTCGTTCT | 57.5 |
|  | *LOC_Os01g08860* | 160 | CTCCGACATCAAGGTGCAGGT | GTTGTCGGGGAGCACGAAC | 27.5 |
|  | *LOC_Os03g16030* | 160 | AGACGGACAAGTGGCACCG | TTGACGTCGGGCTTCTTGGG | 57.5 |
|  | *LOC_Os03g16040* | 160 | TGGACGACGGCAACATCCTG | TCCATGGACGCCTTGATCTGC | 57.5 |
|  | *LOC_Os06g14240* | 160 | GGCGGACCTCTTCTTCGGTG | GTCGTCCTTGCCCAGTCCC | 57.5 |

**Supplementary Table 3.** Correlation analysis for expression values of DEGs between 8 and 15 DAH seeds in NL and RL, respectively.

| NL | Variables | DEGs_8DAH | DEGs_15DAH |
| --- | --- | --- | --- |
|  | DEGs_8DAH | 1.00000 | 0.91802^#^  <0.0001^ |
|  | DEGs_15DAH | 0.91802  <0.0001 | 1.00000 |
| RL | Variables | DEGs_8DAH | DEGs_15DAH |
|  | DEGs_8DAH | 1.00000 | 0.94980  <0.0001 |
|  | DEGs_15DAH | 0.94980  <0.0001 | 1.00000 |

^#^ and ^ indicate correlation coefficient and *p*-value, respectively.

**Supplementary Table 5.** Seed storage protein genes up-regulated in developing rice seeds of Samgwang under salt stress.

| Gene | base Mean | Fold change | *p*-Value | Adjusted *p*-value | Putative Function |
| --- | --- | --- | --- | --- | --- |
| *LOC_Os01g55630* | 19.60 | 3.82 | 1.44E-09 | 4.78E-07 | glutelin |
| *LOC_Os01g55690* | 655329.42 | 1.40 | 0.000860535 | 0.024715898 | glutelin |
| *LOC_Os02g14600* | 42998.04 | 1.13 | 3.41E-07 | 5.23E-05 | glutelin |
| *LOC_Os02g15070* | 9049.57 | 2.68 | 3.61E-08 | 8.34E-06 | glutelin |
| *LOC_Os02g15150* | 254608.16 | 1.38 | 6.57E-10 | 2.53E-07 | glutelin |
| *LOC_Os02g15169* | 10323.04 | 1.69 | 1.80E-09 | 5.74E-07 | glutelin |
| *LOC_Os02g15178* | 6007.57 | 3.47 | 3.73E-07 | 5.58E-05 | glutelin |
| *LOC_Os02g16820* | 52168.81 | 1.86 | 2.47E-05 | 0.001700789 | glutelin |
| *LOC_Os02g16830* | 32927.29 | 1.83 | 8.25E-06 | 0.000710593 | glutelin |
| *LOC_Os03g31360* | 287438.30 | 1.15 | 2.01E-10 | 8.70E-08 | glutelin |
| *LOC_Os05g26240* | 158.16 | 4.71 | 6.90E-05 | 0.003818471 | PROLM1 - Prolamin precursor |
| *LOC_Os05g26350* | 9879.85 | 3.58 | 3.32E-16 | 4.02E-13 | PROLM4 - Prolamin precursor |
| *LOC_Os05g26368* | 10.28 | 4.63 | 5.27E-06 | 0.0004912 | prolamin precursor |
| *LOC_Os05g26440* | 66.75 | 4.89 | 3.13E-13 | 2.35E-10 | PROLM10 - Prolamin precursor |
| *LOC_Os05g26460* | 4682.44 | 4.23 | 8.81E-16 | 9.71E-13 | PROLM11 - Prolamin precursor |
| *LOC_Os05g26480* | 199.26 | 3.95 | 1.98E-14 | 1.92E-11 | PROLM12 - Prolamin precursor |
| *LOC_Os05g26620* | 1920.87 | 6.23 | 3.96E-07 | 5.90E-05 | PROLM14 - Prolamin precursor |
| *LOC_Os05g26690* | 82.11 | 5.71 | 1.10E-06 | 0.000136236 | PROLM15 - Prolamin precursor |
| *LOC_Os05g26720* | 6485.10 | 4.18 | 1.23E-20 | 2.98E-17 | PROLM16 - Prolamin precursor |
| *LOC_Os05g26750* | 4227.20 | 3.65 | 6.59E-12 | 3.90E-09 | PROLM17 - Prolamin precursor |
| *LOC_Os05g26770* | 4403.00 | 4.23 | 4.15E-20 | 9.14E-17 | PROLM18 - Prolamin precursor |
| *LOC_Os07g11900* | 2142.32 | 1.55 | 0.000205802 | 0.008767724 | PROLM19 - Prolamin precursor |
| *LOC_Os07g11910* | 7779.27 | 1.66 | 5.94E-05 | 0.003397122 | PROLM20 - Prolamin precursor |
| *LOC_Os07g11920* | 21470.43 | 2.45 | 4.71E-06 | 0.00045469 | PROLM22 - Prolamin precursor |
| *LOC_Os12g16880* | 135.30 | 4.94 | 4.43E-07 | 6.47E-05 | PROLM27 - Prolamin precursor |
| *LOC_Os12g16890* | 33473.08 | 2.44 | 1.11E-07 | 2.14E-05 | PROLM28 - Prolamin precursor |
| *LOC_Os12g17030* | 1035.39 | 1.53 | 0.000198823 | 0.008575916 | PROLM30 - Prolamin precursor |

**Supplementary Table 7.** Transcription factors selected from DEGs data of the developing seeds of Samgwang at 8 and 15 DAH, respectively.

|  |  | RNA-seq | | | | qRT-PCR | | | |
| --- | --- | --- | --- | --- | --- | --- | --- | --- | --- |
| Transcription  factors | Gene I.D. | 8 DAH | | 15 DAH | | 8 DAH | | 15 DAH | |
|  |  | Log_2_FC^#^ | Adjusted  *p*-value | Log_2_FC | Adjusted  *p*-value | Log_2_FC | *p*-Value | Log_2_FC | *p*-Value |
| NAC | *LOC_Os05g34310* | N.A. | N.A. | 1.05 | 1.85E-08 | 1.47 | 0.0018 | 1.39 | 0.0139 |
| HSF | *LOC_Os08g43334* | N.A. | N.A. | 2.76 | 8.93E-09 | 3.11 | 0.1198 | 1.40 | 0.0081 |
| bZIP1 | *LOC_Os02g43330* | 3.24 | 0.0124 | N.A. | N.A. | 2.59 | 0.0098 | 2.03 | 0.0001 |
| bZIP2 | *LOC_Os07g08420* | 1.42 | 1.20229E-15 | N.A. | N.A. | 2.04 | 0.0002 | -1.93 | 0.0005 |
| bZIP3 | *LOC_Os09g21180* | 2.42 | 1.02053E-05 | N.A. | N.A. | 3.25 | 0.0085 | 1.80 | 0.0005 |
| Zinc finger1 | *LOC_Os03g49730* | N.A. | N.A. | 1.22 | 0.0036 | 1.22 | 0.0069 | 1.04 | 0.0222 |
| Zinc finger2 | *LOC_Os09g26780* | 1.30 | 0.0086 | 1.29 | 0.0008 | 2.25 | 0.0007 | 0.41 | 0.0187 |

^#^: The Log_2_FC (FC, fold change) expresses the Log_2_ value of a ratio of expression value of RL to that of NL (RL/NL), which was generated by RNA-seq and qRT-PCR, respectively, from the developing seeds of Samgwang at 8 and 15 DAH, respectively. The significant difference for each dataset was determined by *t*-test.

**Supplementary Table 8.** Heat shock protein genes selected from DEGs data of the developing seeds of Samgwang at 8 and 15 DAH, respectively.

| Gene I.D. | RNA-seq | | | | qRT-PCR | | | |
| --- | --- | --- | --- | --- | --- | --- | --- | --- |
|  | 8 DAH | | 15 DAH | | 8 DAH | | 15 DAH | |
|  | Log_2_FC^#^ | Adjusted  *p*-value | Log_2_FC | Adjusted  *p*-value | Log_2_FC | *p*-Value | Log_2_FC | *p*-Value |
| *LOC_Os01g04380* | 2.29 | 0.0003 | N.A. | N.A. | 0.85 | 0.0288 | 1.56 | 0.0022 |
| *LOC_Os01g08860* | -1.05 | 0.0016 | 3.30 | 1.53E-40 | -0.59 | 0.0004 | 1.61 | 0.0192 |
| *LOC_Os03g16030* | N.A. | N.A. | 3.01 | 0.0000004 | -0.14 | 0.442 | 1.78 | 0.0007 |
| *LOC_Os03g16040* | N.A. | N.A. | 2.02 | 0.0055 | 0.48 | 0.003 | 1.60 | 0.001 |
| *LOC_Os06g14240* | N.A. | N.A. | 4.77 | 5.87E-08 | 2.01 | 0.0005 | 1.98 | 0.0133 |

^#^: The Log_2_FC (FC, fold change) expresses the Log_2_ value of a ratio of expression value of RL to that of NL (RL/NL), which was generated by RNA-seq and qRT-PCR, respectively, from the developing seeds of Samgwang at 8 and 15 DAH, respectively. The significant difference for each dataset was determined by *t*-test.
